# A Deep Evolution Policy-Based Approach for RIS-Enhanced Communication System

**DOI:** 10.3390/e26121056

**Published:** 2024-12-05

**Authors:** Ke Zhao, Zhiqun Song, Yong Li, Xingjian Li, Lizhe Liu, Bin Wang

**Affiliations:** 1 54th Research Institute of China Electronics Technology Group Corporation, Shijiazhuang 050081, China; zhaoke_sx@163.com (K.Z.); young_li_54@126.com (Y.L.); 18200276858@163.com (X.L.); liu_lizhe@sina.com (L.L.); ctiwangbin@hotmail.com (B.W.); 2 National Key Laboratory of Advanced Communication Networks, Shijiazhuang 050081, China

**Keywords:** reconfigurable intelligent surface (RIS), beamforming, deep evolution policy (DEP), deep neural network (DNN), random subspace selection (RSS)

## Abstract

This paper investigates the design of active and passive beamforming in a reconfigurable intelligent surface (RIS)-aided multi-user multiple-input single-output (MU-MISO) system with the objective of maximizing the sum rate. We propose a deep evolution policy (DEP)-based algorithm to derive the optimal beamforming strategy by generating multiple agents, each utilizing distinct deep neural networks (DNNs). Additionally, a random subspace selection (RSS) strategy is incorporated to effectively balance exploitation and exploration. The proposed DEP-based algorithm operates without the need for alternating iterations, gradient descent, or backpropagation, enabling simultaneous optimization of both active and passive beamforming. Simulation results indicate that the proposed algorithm can bring significant performance enhancements.

## 1. Introduction

Reconfigurable intelligent surface (RIS) represents a promising advancement in the customization of wireless propagation environments [1,2,3]. Specifically, an RIS consists of a planar array comprising a substantial number of reconfigurable reflective elements. Each of these elements is passive and capable of independently inducing a specific phase shift on the incident signal, thereby altering the propagation characteristics of the reflected signal [4,5,6]. However, research concerning the performance analysis and optimization of RIS-aided wireless communication systems is still in its infancy. For example, traditional convex optimization algorithms are no longer suitable because of the non-convex constraint and the mutual coupling between the phase shift and the precoding matrix. Some existing works utilized alternative optimization methods to transform the non-convex problem into a convex optimization problem, such as semi-definite relaxation (SDR) [7], Riemann conjugate gradient (RCG) [8], fractional programming (FP) [9], Majorization-Minimization (MM) [10] and so on. These non-convex optimization algorithms usually require high computational complexity, which is not feasible in wireless communication systems with fast-fading channels and insufficient computing resources. In addition, the solution results depend on the selection of initial values, and the computational complexity increases with the complexity of the communication system, which is inefficient for large-scale systems.

Recently, model-free artificial intelligence (AI) has emerged as a significant technology for addressing the challenges posed by large-scale data, mathematically complex non-linear non-convex problems, and high computational demands. Deep learning (DL) has been applied to RIS-aided communication systems for the purpose of beamforming. In [11], a deep quantization Q network is established to maximize the sum rate, taking into account the discrete quantization of the RIS and the presence of imperfect channel state information (CSI). In [12], a broadband hybrid RIS network is designed based on an RIS-aided terahertz (THz) large-scale multiple-input multiple-output (MIMO) system. The optimization of transmission precoding and the phase shifts of the RIS is conducted jointly through both phase shift and digital networks. To enhance the computational efficiency of RIS-aided hybrid beamforming, a deep learning-based unrolled gradient projection (GP) method is proposed in [13]. This unrolled GP model is trained in a single setting and subsequently tested across thirty-nine different out-of-distribution scenarios. Additionally, ref. [14] examines an RIS-aided frequency division duplex (FDD) massive MIMO downlink system, proposing a DL-based scheme aimed at reducing the overhead associated with CSI feedback by compressing the cascaded CSI. The authors of [15] also investigate the complexities involved in securing unmanned aerial vehicle (UAV)-assisted RIS systems for next-generation communication networks. A deep machine learning framework, specifically the Long Short-Term Memory Deep Deterministic Policy Gradient (LSTM-DDPG), is proposed to effectively address security concerns and ensure reliable communication within UAV-assisted RIS networks by mitigating potential malicious threats. DL models are traditionally dense and over-parameterized, sometimes to the extent that they can memorize random patterns in the data or that 95% of the parameters can be predicted from the remaining 5%. Sparsity can reduce the memory footprint of regular networks to fit mobile devices, as well as shorten the training time for ever-growing networks [16]. The authors of [17] introduce a Sparsely-Gated Mixture-of-Experts (MoE) layer, which consists of a large number of feed-forward neural network “experts” and a trainable gating network that selects a sparse combination of these experts to process each input. In order to reduce the complexity of the Transformer model, ref. [18] introduces BIGBIRD, a sparse attention mechanism that reduces the quadratic dependency on sequence length in Transformer models to being linear, which also shows promise for novel applications in genomics data. The authors of [19] introduce a novel model-based perspective on machine unlearning (MU), which aims to remove the influence of specific training examples from a machine learning model. The key insight is that model sparsification via weight pruning can significantly reduce the gap between exact unlearning and approximate unlearning.

Different from DL, deep reinforcement Learning (DRL) is an online training algorithm that embraces the advantage of DL in neural network training as well as improving the learning speed and performance of reinforcement learning (RL) algorithms. A standard DRL optimization framework is established in [20], which is based on an RIS-assisted MU-MISO communication system. This framework allows for the simultaneous optimization of both active and passive beamforming with minimal implementation complexity. In [21], a DRL algorithm is proposed to jointly optimize beamforming by developing a position-sensing model that reduces interactive overhead. In [22], an RIS-aided downlink orthogonal frequency division multiplexing (OFDM) transmission system is investigated, which employs the twin delayed deep deterministic policy gradient (TD3) method for optimizing RIS phase shifts to minimize the transmission power. In [23], the authors introduce a Soft Actor-Critic (SAC) algorithm aimed at jointly optimizing transmit beamforming and RIS phase shifts within a MU-MISO system, with the objective of maximizing the sum downlink rate under a phase-dependent reflection amplitude model. Ref. [24] explores a downlink OFDM transmission system aided by RIS, proposing a DRL-based optimization algorithm that utilizes a deep Q-network (DQN) framework to enhance spectral efficiency. In [25], a novel approach derived from the deep deterministic policy gradient (DDPG) algorithm, specifically the Shared Deep Deterministic Policy Gradient (SD-DDPG) algorithm, is introduced to address the power allocation and trajectory optimization challenges for downlink unmanned aerial vehicle (UAV)-mobile instruments (MIs). In [26], the simultaneous transmitting and reflecting reconfigurable intelligent surface (STAR-RIS) assisted non-orthogonal multiple access (NOMA) system is investigated, which includes a cooperative jammer and dual eavesdroppers. The DDPG framework is employed to maximize the sum secrecy rate by jointly optimizing channel allocation, transmit power, and coefficient matrices under both perfect and imperfect CSI.

However, gradient descent-based AI algorithms, such as DL and DRL, require the minimization of a loss function, necessitating that the optimization model be differentiable. Additionally, the development of an agent with appropriate hyperparameters can pose significant challenges. In recent years, metaheuristic algorithms have been employed to address complex real-world problems. The challenges that emerge from various domains, including economics, engineering, politics, and management, are significant [27,28,29]. In [30], an innovative genetic locus selection scheme is introduced, which serves as the basis for parameter configuration of the crossover operator by formulating the process as a Markov Decision Process (MDP). Ref. [31] examines an uplink RIS-aided massive MIMO system, focusing on the optimization of phase shifts at the RIS through a genetic algorithm (GA) to maximize both the sum rate and the minimum user rate. Furthermore, ref. [32] explores the performance of RIS-aided massive MIMO systems that incorporate direct links, with the phase shifts of the RIS being designed via GA based on statistical CSI.

The aforementioned works primarily integrate evolution learning (EL) and RL, wherein individuals are represented by discrete vectors. However, the use of parameter vectors may limit the expression of complex features, potentially resulting in a reduction in diversity within the population. Inspired by the benefits of EL and DRL, we propose an algorithm based on deep evolution policy (DEP). Differently from model-based schemes, which rely on gradient descent and backpropagation processes within one single deep neural network (DNN), the proposed DEP-based algorithm generates multiple individuals with diverse characteristics that interact with the environment within a single generation. Specifically, we employ various DNNs to represent different individuals within the population. Following the processes of selection, crossover, and mutation, a new generation comprising multiple individuals will continue to interact with the environment until the performance of the entire population converges. Therefore, the active beamforming at the base station (BS) and the passive beamforming at the RIS can be optimized through the individual with the highest score in the last generation. Furthermore, without processes such as gradient descent and backpropagation, the DEP-based approach can significantly decrease the computation complexity. Ultimately, simulations are conducted to validate our analytical findings.

## 2. System Model and Problem Formulation

**Figure 1:**
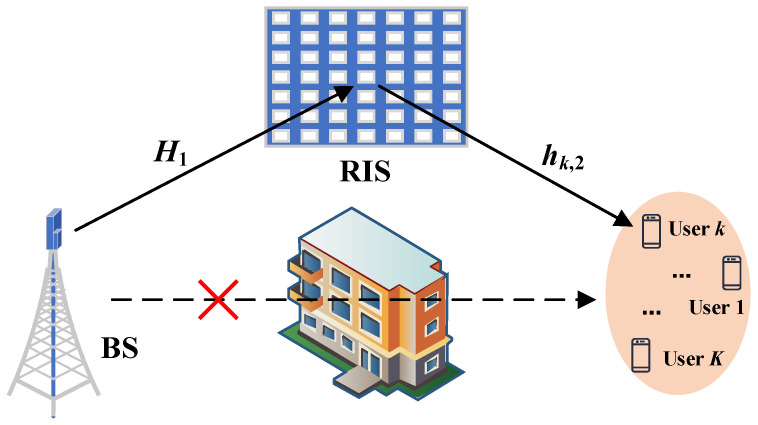
The considered RIS-aided MU-MISO communication system comprised of an *M*-antenna BS simultaneously serving in the downlink *K* single-antenna users, and the transmit signal propagates to the users via the RIS assistance.

As shown in Figure 1, we consider an RIS-aided downlink MU-MISO communication system where an RIS is deployed to provide high-quality virtual links from the multi-antenna BS to *K* single-antenna users. The number of transmit antennas at the BS and the number of reflecting elements at the RIS are denoted by *M* and *N*, respectively. The baseband equivalent channels from the BS to the RIS and from the RIS to the user *k* are denoted by $H_{1}\in C^{N\times M}$ and $h_{k,2}\in C^{N\times 1}$, respectively, and the direct channel is blocked.

The complex baseband transmitted signal at the BS can be expressed as $x=\sum_{k=1}^{K}\omega_{k}s_{k}$, where $s_{k}$ denotes the transmission symbol for the user *k* with normalized power (zero mean and unit variance) and $\omega_{k}\in C^{M\times 1}$ is the corresponding beamforming vector. The signal received at the user *k* can be expressed as

(1) $$y_{k}=h_{k,2}^{H}\Phi H_{1}\sum_{j=1}^{K}\omega_{j}s_{j}+n_{k},k=1,\ldots ,K,$$

where $\Phi =\mathrm{diag}\{e^{j\theta_{1}},e^{j\theta_{2}},...,e^{j\theta_{N}}\}$ with unit reflection amplitude denoting the configuration matrix, and $\theta_{n}$ representing the phase shift of element *n*. $n_{k}∼CN\left(0,\sigma_{k}^{2}\right)$ denotes the additive white Gaussian noise (AWGN) at the receiver of the user *k*. In a multi-user system, the user *k* treats all the signals from other users as interferences. Hence, the signal-to-interference-noise ratio (SINR) $\gamma_{k}$ at the user *k* can be expressed as

(2) $$\gamma_{k}=\frac{{\left|h_{k,2}^{H}\Phi H_{1}\omega_{k}\right|}^{2}}{\sum_{\begin{array}{c} m=1\\ m\neq k \end{array}}^{K}{\left|h_{k,2}^{H}\Phi H_{1}\omega_{m}\right|}^{2}+\sigma_{k}^{2}}.$$

In this paper, our objective is to maximize the system sum rate of all users by jointly designing the active and passive beamforming. The optimization problem is formulated as

(3) $$\begin{array}{lll} \left(P1\right): & \max_{W,\Phi } & f\left(W,\Phi \right)=\sum_{k=1}^{K}\log_{2}\left(1+\gamma_{k}\right)\\ & \;s.t.\;\; & C1:\left|\theta_{n}\right|=1,n=1,\ldots ,N,\\ & \; & C2:\mathrm{Tr}\left(W^{H}W\right)\leq P_{t}, \end{array}$$

where $W={\left\{\omega_{k}\right\}}_{k=1}^{K}$ and $P_{t}$ represent the active beamforming matrix and transmit power at the BS, respectively.

## 3. Algorithm Description

### 3.1. Overview of DRL

In a typical RL process, the agent incrementally determines the optimal action through interactions with the environment. The agent can observe instant rewards and state transitions in the environment by executing actions. Specifically, RL can be formulated as an MDP, which encompasses several fundamental components that comprehensively characterize the RL learning process: the state, the action, and the instant reward.

**State**: observations of the environment. The state $s^{\left(t\right)}\in S$ denotes the observation at the time step t.

**Action**: choices of the agent. The agent takes one action step by step during the learning process. Once the agent takes an action $a^{\left(t\right)}\in A$ at time instant *t* following a policy π, the state of the environment will transit from the current state $s^{\left(t\right)}$ to the next state $s^{\left(t+1\right)}$.

**Reward**: return of the action. By taking action $a^{\left(t\right)}$ in a given state $s^{\left(t\right)}$, the agent will obtain a reward $r^{\left(t\right)}$, which is a performance metric to evaluate how good the action is.

The MDP can be represented as a tuple (S,A,P,γ), where *P* is the transition dynamics such that $s^{\left(t+1\right)},r∼P\left(s^{\left(t\right)},a^{\left(t\right)}\right)$ and γ∈[0,1] is a discount factor which prioritizes the short-term rewards. The purpose of RL is to find the optimal policy π that can maximize the total reward defined by $V_{t}=\sum_{i=0}^{\infty }\gamma^{i}r_{t+i+1}$. The policy can be regarded as stochastic π:S→p(A) or deterministic π:S→A. By adopting the Q-function as the state-action value function, the Bellman equation can be expressed as follows:

(4) $$Q^{\pi }\left(s^{\left(t\right)},a^{\left(t\right)}\right)=E_{r^{\left(t\right)},s^{\left(t+1\right)}∼P,a^{\left(t+1\right)}∼\pi }\left[r^{\left(t\right)}+\gamma Q^{\pi }\left(s^{\left(t+1\right)},a^{\left(t+1\right)}\right)\right],$$

where $a^{\left(t+1\right)}$ is the action selected by the policy on the next state $s^{\left(t+1\right)}$.

In a typical DRL, the critic is usually approximated by a deep neural network $Q_{\theta }$, where θ is the parameters of the DNN. For training $Q_{\theta }$, a temporal-difference (TD) algorithm is adopted to minimize the loss J(θ) with TD error δ, which is the difference between the output of $Q_{\theta }$ and the learning target *y*.

(5) $$y^{\left(t\right)}≜r^{\left(t\right)}+\gamma Q_{\theta^{\left(t+1\right)}}\left(s^{\left(t+1\right)},a^{\left(t+1\right)}\right),$$

(6) $$\delta ≜y^{\left(t\right)}-Q_{\theta }^{\left(t\right)}\left(s^{\left(t\right)},a^{\left(t\right)}\right),$$

(7) $$\theta \leftarrow \theta -\eta \nabla_{\theta }J\left(\theta \right),$$

where $J\left(\theta \right)=|\delta {|}^{2}$, $\nabla_{\theta }J\left(\theta \right)$ is the gradient of the loss J(θ) with respect to θ, and η is the learning rate.

### 3.2. Deep Evolution Policy

**Figure 2:**
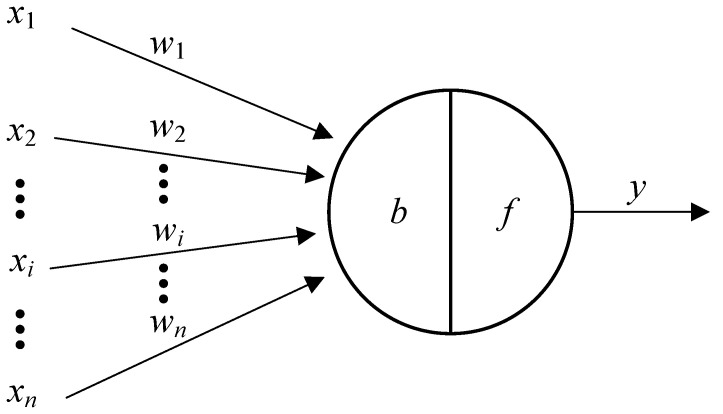
M-P neuron model.

A typical M-P neuron model is illustrated in Figure 2, where $x_{i}$ represents the *i*-th input signal from other neurons. $\omega_{i}$, *b*, and *f* denote the weight, bias, and activation function, respectively. The output of the M-P neuron can be expressed as follows:

(8) $$y=f\left(\sum_{i=1}^{n}\omega_{i}x_{i}-b\right)$$

**Figure 3:**
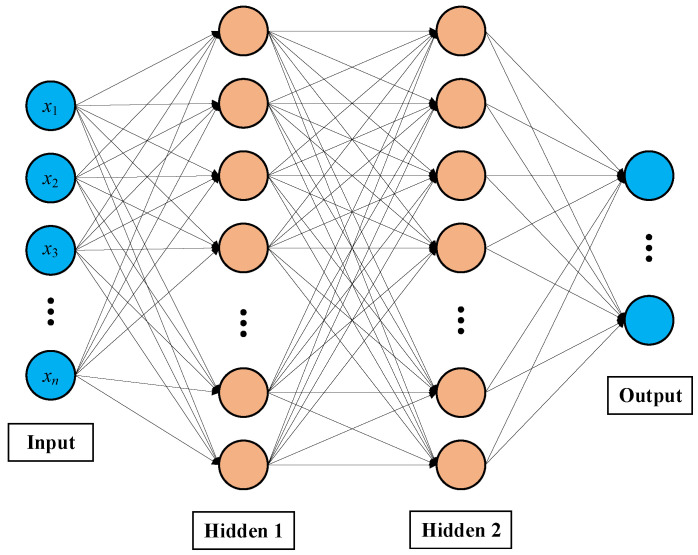
DNN model with two hidden layers.

The limited learning capacity of a single neuron necessitates the adoption of DNNs, which comprise multiple layers of neural networks, to effectively address nonlinear separable problems. Typically, the process of DL can be represented as nonlinear function approximation, wherein multi-layer neural networks decompose complex mappings into a series of simpler mappings, each represented by different layers of the model. DRL is an RL algorithm that leverages the robust nonlinear fitting capabilities of DL to optimize the parameters of neural networks through interactions with the environment. Additionally, DEP is an evolutionary learning algorithm that treats distinct DNNs as individual entities within a population. A DNN featuring two hidden layers is illustrated in Figure 3.

**Figure 4:**
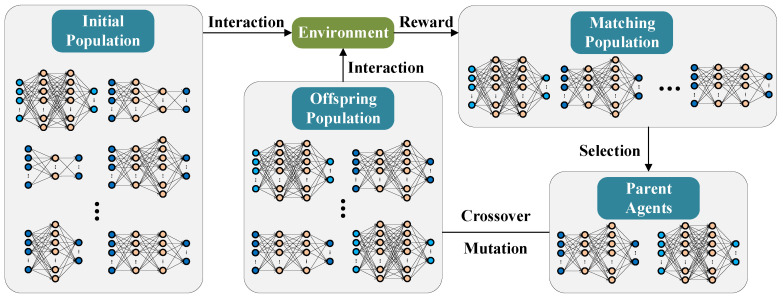
The schematic diagram of the DEP-based algorithm, which includes the processes of interaction, selection, crossover and mutation.

The proposed DEP-based algorithm integrates the advantages of RL and DL by employing an evolutionary method inspired by unnatural selection. Specifically, the natural selection process inherent to biological evolution is simulated through the use of DEP in conjunction with DNNs and evolutionary algorithms. In each generation, multiple deep neural networks with varying structures and parameter vectors are concurrently generated, which then interact with the environment to yield diverse fitness scores. The environment may be regarded as a fitness function, with the fitness score serving as a reward according to the current state and characteristics of the agents (individuals). During the evolutionary process, agents that yield higher rewards are typically favored in the selection process. The schematic diagram of the DEP-based algorithm is illustrated in Figure 4.

1. Initialize a population that includes *P* individuals, each consisting of deep natural networks with different parameters and structures. Each DNN can be considered an agent;
2. Calculate the fitness score of each agent through interacting with the environment, subsequently obtaining the matching population of agents along with their respective rewards;
3. Select the parent agents from the matching population according to a specific selection strategy;
4. *P* offspring agents are generated from parent agents through a crossover process. Additionally, a mutation process is implemented to promote genetic diversity;
5. The new population continues to engage with its environment until it achieves a state of convergence, where the average fitness of the population stops showing a notable enhancement.

### 3.3. Random Subspace Selection

The objective of RL is to identify the optimal strategy by leveraging the available information, which can only be acquired through exploration. Consequently, it is crucial to achieve a balance between exploration and exploitation. Two extreme baseline selection strategies are the pure exploitation strategy and the pure exploration strategy. The pure exploitation strategy, often referred to as the greedy strategy, ranks all agents based on their rewards and selects the two agents with the highest rewards as the parent agents. Conversely, the pure exploration strategy, commonly known as the random strategy, selects parent agents at random. Most existing selection strategies integrate both the pure exploitation and pure exploration strategies, with typical mixed selection strategies including the soft-max strategy, ϵ-greedy strategy, and attenuated ϵ-greedy strategy.

Most mixed selection strategies emphasize the accumulation of experience during the initial stages and the exploration of actions in the later stages. In contrast to gradient descent-based algorithms, the DEP-based algorithm needs more extensive exploration space for generating offspring through population reorganization and mutation in each generation. In this paper, we propose a random subspace selection (RSS) strategy for the selection of parent agents, as summarized in Algorithm  1. Specifically, Pη agents are randomly selected from the matching population to form the subspace population, where η represents the proportion of the subspace. The agents within the subspace population are ranked according to their fitness scores, and parent agents are selected based on the highest fitness scores. Notably, (1) when η=1, the subspace encompasses the entire population, resulting in the RSS degenerating into a pure exploitation strategy, and (2) when η=2/P, the subspace consists of only two agents, leading to the RSS degenerating into a pure exploration strategy. Consequently, the appropriate selection of the subspace proportion η can effectively balance exploration and exploitation.
**Algorithm 1** Random Subspace Selection Strategy.1:**Input:** matching population, *P*.2:Initialize: η,m=1.3:**Repeat:**4:     Random select agent from matching population.5:      m=m+1.6:**Until:** m=Pη7:Select the agents with two highest fitness scores as parents from *m* agents.8:**Output:** parent agents.

## 4. Joint Active and Passive Beamforming Optimization Based on DEP

The RIS-aided MU-MISO downlink communication system is considered in this paper, and the active beamforming at the BS and the passive beamforming at the RIS are jointly optimized with the DEP-based algorithm. The initial population size, the matching population size, and the offspring population size are all set as *P* in *G* generations. A few basic elements of the DEP-based algorithm are shown as follows:

**State**: Different from gradient descent-based DRL algorithms, the neural network structure is optimized without backpropagation in the DEP-based algorithm. Thus, the state $s^{\left(t\right)}$ only includes the channels $H_{1}^{\left(t\right)}$ and $h_{k,2}^{\left(t\right)}$ at the time step *t*. H1=Re{H1}+Im{H1}, hk,2=Re{hk,2}+Im{hk,2}. The real part and the imaginary part of each entry of $H_{1}$ and $h_{k,2}$ are also used as entries of the state, and the dimension of the state space is 2NM+2NK.

**Action**: After receiving the input state $s^{\left(t\right)}$, the deep natural network which represents the agent outputs the action $a^{\left(t\right)}$. The action is simply constructed by the transmit beamforming matrix $W^{\left(t\right)}$ and the phase shift matrix $\Phi^{\left(t\right)}$. Likewise, to tackle the real input problem, W=Re{W}+Im{W} and Φ=Re{Φ}+Im{Φ} are separated as a real part and imaginary part, and both are entries of the action. The dimension of the action space is 2MK+2N.

**Reward**: The reward $r^{\left(t\right)}$, that is the system sum-rate, is produced by the interaction between the action $a^{\left(t\right)}$ and the fitness function as shown in (9).

(9) $$r^{t}=f\left(W^{t},\Phi^{t}\right)=\sum_{k=1}^{K}\log_{2}\left(1+\gamma_{k}\right).$$

The DNN utilized in this paper is characterized by a uniform network architecture, wherein each agent is represented as a parameter vector comprising distinct weights *w* and biases *b*. It is assumed that both hidden layers have the same dimension $h_{\dim}$. Consequently, all agents can be expressed as ${\mathrm{Para}}_{p}\left(p=1,2,\ldots ,P\right)$ with a consistent dimensional size of *D*, where $D=2\left(NM+NK\right)h_{\dim}+\left(h_{\dim}+2\right)h_{\dim}+2\left(MK+N\right)h_{\dim}+2\left(MK+N\right)$.

**Figure 5:**
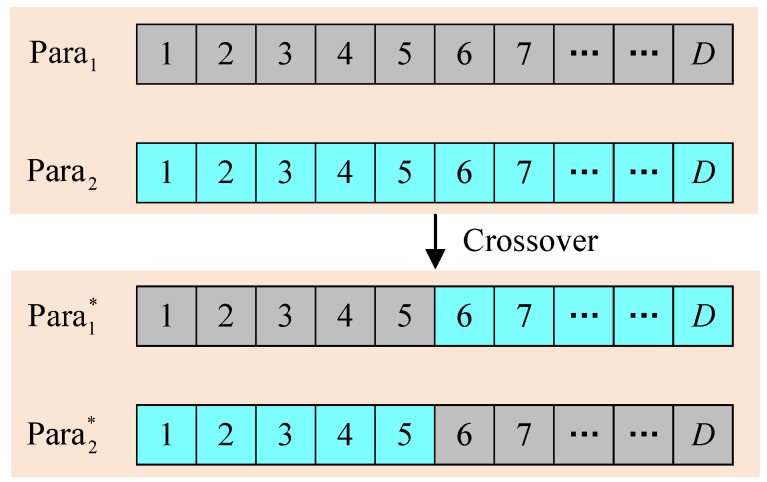
Crossover process. The boxes in gray and cyan represent the genetic information of ${\mathrm{Para}}_{1}$ and ${\mathrm{Para}}_{2}$, respectively.

Figure 5 shows the crossover process between parent agents. Specifically, parent agents ${\mathrm{Para}}_{1}$ and ${\mathrm{Para}}_{2}$ choose a random genetic locus to crossover, and then produce offspring agents ${\mathrm{Para}}_{1}^{*}$ and ${\mathrm{Para}}_{2}^{*}$.

(10) $$\left({\mathrm{Para}}_{1}^{*},{\mathrm{Para}}_{2}^{*}\right)=\mathrm{cross}\left({\mathrm{Para}}_{1},{\mathrm{Para}}_{2}\right),$$

where cross(·) denotes the crossover process. The parent agents will continue to execute the crossover process until the offspring population reaches the size of *P*.

**Figure 6:**
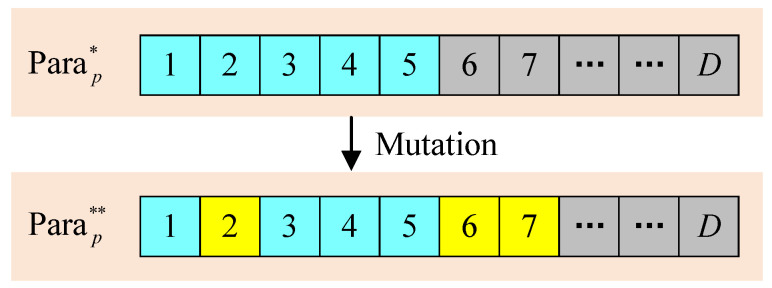
Mutation process. The boxes in gray, cyan and yellow represent the genetic information of ${\mathrm{Para}}_{1}$, ${\mathrm{Para}}_{2}$, and mutation respectively.

Figure 6 illustrates the mutation process designed to reduce the risk of the population converging to local optima. The primary aim of the mutation process is to incorporate new genetic information into the population.

(11) $${\mathrm{Para}}_{p}^{**}=\mathrm{mut}\left({\mathrm{Para}}_{p}^{*},\mu \right)$$

where mut(·) represents the mutation process, while μ represents the individual mutation rate. Specifically, agent *p* with mutation rate μ randomly selects μD parameters for mutation from the set ${\mathrm{Para}}_{p}^{*}$. It is important to note that agents with high mutation rates may cause the most fit individuals to lose advantageous genetic information. Conversely, agents with low mutation rates may struggle to identify the optimal policy due to the limited introduction of new information. Therefore, selecting an appropriate mutation rate is crucial for the DEP algorithm. Consequently, *P* agents characterized by varying parameter vectors can be generated within the new population following the crossover and mutation processes. As the generation number increases to *G*, the agent exhibiting the highest reward can be decomposed into distinct weights *w* and *b*, which can subsequently be utilized to construct a new DNN. The proposed DEP-based approach is summarized as Algorithm  2.
**Algorithm 2** DEP-based algorithm.  1:**Input:** $H_{1}^{\left(t\right)},h_{k,2}^{\left(t\right)},k=1,2,\ldots ,K.$  2:Initialize: P,G,μ,g=1.  3:Generate *P* agents for the initial population with the dimension size of *D*.  4:**Repeat:**  5:     Produce matching population by (9).  6:     Select ${\mathrm{Para}}_{1}$ and ${\mathrm{Para}}_{2}$ through Algorithm  1.  7:     **Repeat:**  8:          Perform crossover process by (10).  9:          Perform mutation process by (11).10:     **Until:** The offspring population size = *P*.11:      g=g+1.12:**Until:** g=G13:Construct a DNN utilizing the agent with the highest reward.14:Calculate $W^{\left(t\right)}$ and $\Phi^{\left(t\right)}$ through the proposed DNN.15:**Output:** $W^{\left(t\right)},\Phi^{\left(t\right)}$.

Assume that the generation number of the DEP algorithm is *G*, and each generation consists of *P* individuals. The computational complexity of the DEP mainly comes from the fitness evaluation in each generation, and the complexity of matching, crossover, and mutation can be neglected. From (2), the computational complexity of the fitness evaluation is $O\left(KN\right)$, and the proposed DEP has an approximate computational complexity of $O\left(GPKN\right)$. In addition, the computational complexity of the DDPG and SAC algorithms mainly comes from the fitness evaluation and the network updates, and the target network updating and the experiment reply can be neglected. Assume that the batch size and the number of network parameters are *B* and *D*, respectively. With *T* time steps, the computational complexity of DDPG and SAC is $O\left(T(BD+KN)\right)$

## 5. Numerical Results

In this section, we present numerical simulations to evaluate the proposed DEP algorithm. We selected two state-of-art DRL algorithms as benchmarks: the DDPG algorithm [20], and the SAC algorithm [23]. The hyperparameter settings are detailed in Table 1 and Table 2. It is important to distinguish between episodic and non-episodic tasks within the context of the environments. An episodic task concludes when a terminal condition is met, whereas a non-episodic (continuing) task does not have a specific endpoint. In this paper, the communication environment is classified as continuing, as the BS continuously performs beamforming and configures RIS elements. Consequently, we have set the discount term to 1, as the agent must equally prioritize both instant and future rewards, given that the objective is to maximize the cumulative reward (sum rate).

From Figure 1, we can see that the direct channel from the BS to the User is blocked, and the transmission signal is transmitted through the reflection line-of-sight (LoS) links $H_{1}$ and $h_{k,2}$. $H_{1}$ and $h_{k,2}$ follow the Rician fading which can be modeled by

(12) $$H_{1}=L_{1}\left(\sqrt{\frac{K_{1}}{K_{1}+1}}{\bar{H}}_{1}+\sqrt{\frac{1}{K_{1}+1}}{\tilde{H}}_{1}\right),$$

(13) $$h_{k,2}=L_{2}\left(\sqrt{\frac{K_{2}}{K_{2}+1}}{\bar{h}}_{k,2}+\sqrt{\frac{1}{K_{2}+1}}{\tilde{h}}_{k,2}\right),$$

where $L_{1}$ and $L_{2}$ denote the large-scale path losses, which can be modeled by 32.4 + 20lg(*f*) + 19.8lg(*d*) + 2 according to the 3GPP propagation environment. *f* is the signal frequency, and *d* is the distance between the transceiver. In this paper, the carrier frequency, the transmission bandwidth, and the noise power spectral density are set at 3.5 GHz, 200 kHz, and −170 dBm/Hz, respectively. $K_{1}$ and $K_{2}$ are the Rician factors. It can be seen that when the Rician factor is 0, the reflect link becomes a non-line-of-sight (NLoS) channel which follows the Rayleigh fading, and we set $K_{1}=K_{2}=10$ for simulation. ${\tilde{H}}_{1}$ and ${\tilde{h}}_{k,2}$ denote NLoS components whose elements are chosen from $CN\left(0,1\right)$, while ${\bar{H}}_{1}$ and ${\bar{h}}_{k,2}$ denote LoS components whose elements consist of the antenna array responses of the transmitter and receiver.

**Figure 7:**
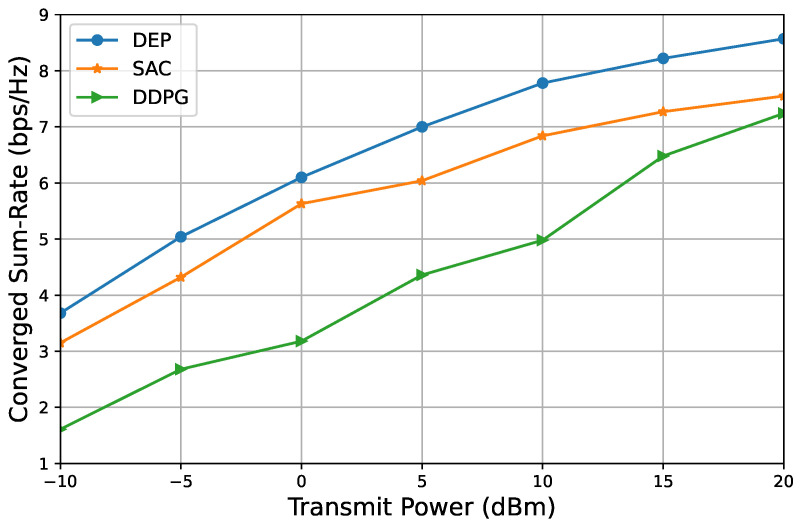
Converged sum rate versus transmit power to show the proposed DEP-based algorithm in comparison with two benchmarks, M=4,N=4,K=4.

**Figure 8:**
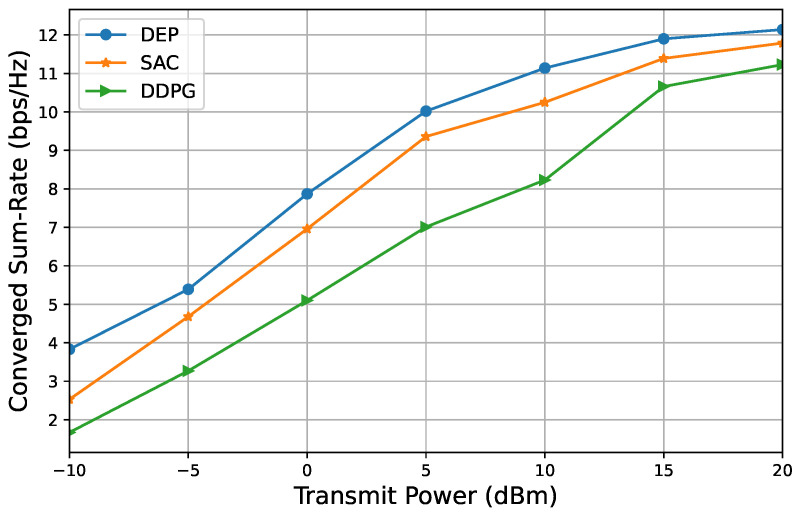
Converged sum rate versus transmit power to show the proposed DEP-based algorithm in comparison with two benchmarks, M=4,N=32,K=4.

We have conducted an evaluation of the proposed DEP-based approach, as outlined in Algorithm 2, in comparison to two benchmark methods. Figure 7 and Figure 8 illustrate the relationship between the converged sum rate and transmit power. Two sets of system parameters were considered: M=4,N=4,K=4, and M=4,N=32,K=4. The results indicate that the performance of our proposed DEP-based approach surpasses that of the state-of-the-art benchmarks, specifically the DDPG and SAC algorithms. Furthermore, it is observed that the converged sum rate increases with transmit power across all the considered algorithms and scenarios.

**Figure 9:**
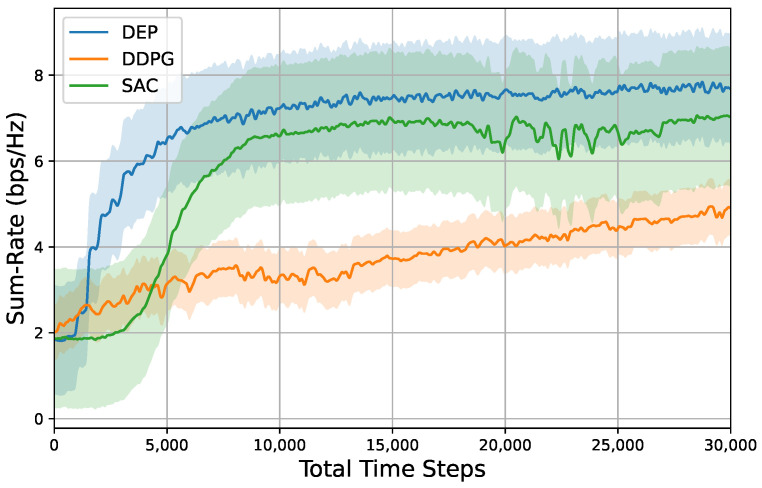
Sum rate as a function of total time steps at $P_{t}=10\;\mathrm{dBm},M=4,N=4,K=4$. Shaded regions represent 95% confidence interval over 10 random seeds for each result.

Figure 9 illustrates the convergence behavior of our proposed DEP-based algorithm in comparison to the DDPG and SAC algorithms. After approximately 10,000 time steps, the system sum rate of the SAC algorithm converges to 6.7 bps/Hz. In contrast, the sum rate of the DDPG algorithm reaches approximately 4.8 bps/Hz after 30,000 time steps. The slower convergence of the DDPG algorithm can be attributed to the continuous interactions between a single agent and the environment. Additionally, the actions generated by the DDPG algorithm exhibit limited exploration due to their deterministic nature. The SAC algorithm incorporates an additional critic network compared to DDPG. To enhance exploration within the actor network, the SAC algorithm employs an entropy-based method. In contrast to these DRL algorithms, our proposed DEP-based approach facilitates the simultaneous interactions between multiple agents and the environment. Notably, after approximately 10,000 steps, equivalent to 20 generations (with a population size of 500 for each generation), the average reward converges to 7.3 bps/Hz. It is important to highlight that the final policy of a DRL algorithm depends on the last agent that interacts with the environment, while the DEP-based approach incorporates multiple agents in the final generation. Due to the robust exploration capabilities of the DEP algorithm, the agent yielding the highest instant reward can be selected as the final policy in the last generation. Consequently, the optimal sum rate achievable through the DEP algorithm can reach approximately 9 bps/Hz.

**Figure 10:**
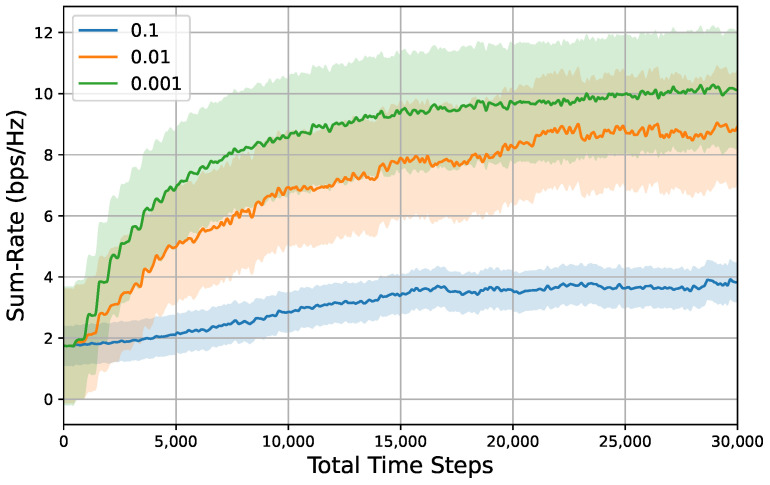
Sum rate versus total time steps under different mutation rates, i.e., {0.1,0.01,0.001}, $P_{t}=5\;\mathrm{dBm},M=4,N=32,K=4$, population size = 500, subspace proportion = 0.5. Shaded regions represent 95% confidence interval over 10 random seeds for each result.

**Figure 11:**
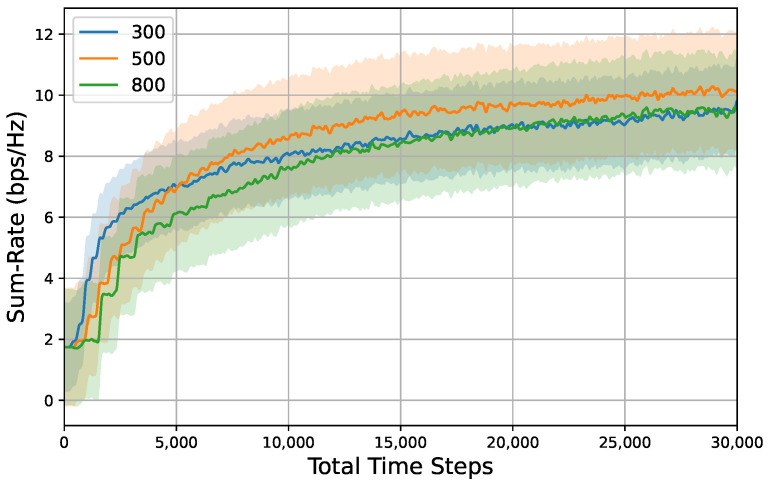
Sum rate versus total time steps under different population sizes, i.e., {300,500,800}, $P_{t}=5\;\mathrm{dBm},M=4,N=32,K=4$, mutation rate = 0.001, subspace proportion = 0.5. Shaded regions represent 95% confidence interval over 10 random seeds for each result.

**Figure 12:**
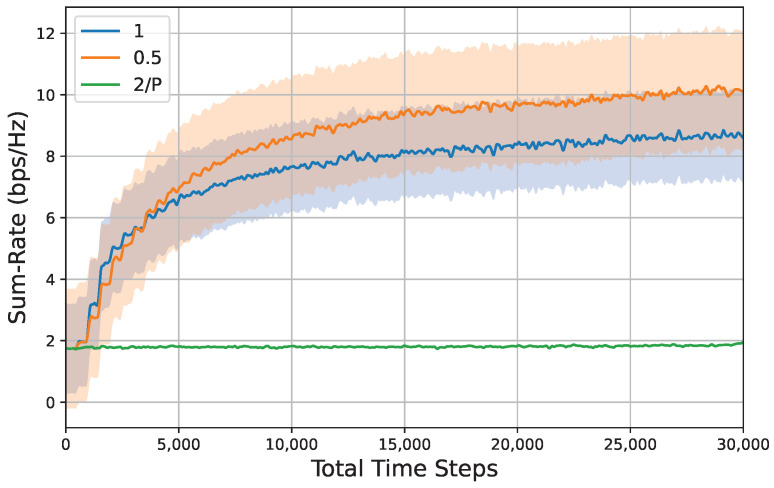
Sum rate versus total time steps under different subspace proportions, i.e., {2/P,0.5,1}, $P_{t}=5\;\mathrm{dBm},M=4,N=32,K=4$, mutation rate = 0.001, population size = 500. Shaded regions represent 95% confidence interval over 10 random seeds for each result.

To investigate the influence of hyperparameter settings on system performance, we established several groups of comparative simulations that included variations in mutation rate, population size, and subspace proportion. The results of these simulations are presented in Figure 10, Figure 11 and Figure 12.

Figure 10 demonstrates the sum rate versus time steps under different mutation rates. The data indicate that mutation rates significantly affect the performance of the DEP algorithm. Specifically, the system achieves optimal performance in terms of converged sum rate at a mutation rate of 0.001. Conversely, an increase in the mutation rate to 0.1 results in a deterioration of the sum rate convergence. This convergence behavior can be attributed to the loss of advantageous genetic information within the population when subjected to a high mutation rate. Furthermore, it is important to highlight that excessively low mutation rates may lead the population to become trapped in local optima due to the insufficient introduction of new genetic material. In conclusion, it is essential to select an appropriate mutation rate that aligns with environmental parameters, ensuring it is neither excessively high nor excessively low.

Figure 11 illustrates the relationship between the sum rate and time steps across varying population sizes. The results indicate that the DEP algorithm with a population size of 500 exhibits an optimal performance for the parameters M=4,N=32,K=4. While smaller population sizes demonstrate a more rapid convergence rate, they are prone to becoming trapped in local optima due to insufficient population diversity. Conversely, excessively large population sizes result in slower convergence rates and may also lead to local optima, as they tend to contain a high number of individuals with similar characteristics.

Figure 12 illustrates the relationship between sum rate and time steps across various subspace proportions, thereby highlighting the impact of the parent agent selection process as outlined in Algorithm 1. In this context, it is necessary to select two agents from a population consisting of *P* agents to serve as parents, followed by the execution of crossover and mutation processes to generate an offspring population. Consequently, the strategy employed for selecting parent agents is of paramount importance. It can be seen that when the population size is *P*, the subspace proportion of 2/P corresponds to a random selection of parent agents. This random selection implies that the offspring may not effectively inherit the dominant genes, resulting in no significant improvement in system performance. Moreover, when the subspace proportion is set to 1, the selection of parent agents necessitates a comprehensive traversal of the population to identify the two individuals with the highest fitness scores. This exhaustive search can lead to a lack of randomness in the hereditary process, thereby increasing the likelihood of the population becoming trapped in local optima during the iterative process.

## 6. Conclusions

In this paper, we investigate an RIS-aided MU-MISO downlink communication system. We propose a DEP-based algorithm for the joint optimization of both active and passive beamforming. Unlike optimizing one single DNN through gradient descent-based algorithms, the proposed DEP-based approach simultaneously generates multiple agents composed of DNNs in one population. After the processes of selection, crossover, and mutation, the active and passive beamforming can be optimized through the agent with the highest score in the last generation. This approach eliminates the need for gradient backpropagation and optimization for individual agents, allowing for the concurrent calculation of fitness scores for multiple agents. Furthermore, it facilitates the processes of selection, crossover, and mutation to produce offspring with enhanced characteristics. In conclusion, we propose a new way to solve beamforming optimization with faster convergence speed and higher system performance. Ultimately, the simulation results demonstrate the effectiveness and superiority of the DEP-based method in comparison to the DRL algorithm that relies on gradient descent.

## Figures and Tables

**Table 1:** DRL-based algorithm hyperparameter settings.

Parameter	Value
Optimizer	Adam
Total time steps per training	30,000
Experience replay buffer size	20,000
Mini-batch size	16
Network update interval	after each environment step
Discount term	1
Learning rate for training critic network	0.001
Learning rate for training actor network	0.001
Learning rate for target critic network	0.001
Learning rate for target actor network	0.001
Entropy target	-action dimension
SAC log standard deviation clipping	(−20, 2)
SAC ϵ	$10^{-6}$

**Table 2:** DEP-based algorithm hyperparameter settings.

Parameter	Value
Population size *P*	500
Subspace proportion η	0.5
Mutation rate μ	0.001
Generation *G*	60

## Data Availability

All data generated or analyzed during this study are included in this published article.
